# Prevention of Death by Chloroform

**Published:** 1856-04

**Authors:** 


### THE PREVENTION OF DEATH BY CHLOROFORM.*

Dr. Snow states as an absolute fact, that all the accidents which have occurred during the exhibition of chloroform, have been caused by the air breathed by the patient being at some moment too highly charged with the narcotic vapour; so that, to prevent such accidents, it is only necessary to ensure that the vapour shall at all times be sufficiently diluted with air. In commencing the inhalation, the patient should take in a vapour diluted with not less than 95 per cent. of air. Artificial respiration, promptly applied, is the only measure which promises success in cases of accident from chloroform.

* Further Remarks on the Prevention of Death by Chloroform. By John Snow, M.D. London: 1856.

